# Gaze Strategies in Driving–An Ecological Approach

**DOI:** 10.3389/fpsyg.2022.821440

**Published:** 2022-03-14

**Authors:** Otto Lappi

**Affiliations:** Cognitive Science/TRU, University of Helsinki, Helsinki, Finland

**Keywords:** locomotor control, visual guidance, guiding fixations, look-ahead fixations, pursuit eye movement, ecological psychology

## Abstract

Human performance in natural environments is deeply impressive, and still much beyond current AI. Experimental techniques, such as eye tracking, may be useful to understand the cognitive basis of this performance, and “the human advantage.” Driving is domain where these techniques may deployed, in tasks ranging from rigorously controlled laboratory settings through high-fidelity simulations to naturalistic experiments in the wild. This research has revealed robust patterns that can be reliably identified and replicated in the field and reproduced in the lab. The purpose of this review is to cover the basics of what is known about these gaze behaviors, and some of their implications for understanding visually guided steering. The phenomena reviewed will be of interest to those working on any domain where visual guidance and control with similar task demands is involved (e.g., many sports). The paper is intended to be accessible to the non-specialist, without oversimplifying the complexity of real-world visual behavior. The literature reviewed will provide an information base useful for researchers working on oculomotor behaviors and physiology in the lab who wish to extend their research into more naturalistic locomotor tasks, or researchers in more applied fields (sports, transportation) who wish to bring aspects of the real-world ecology under experimental scrutiny. Part of a Research Topic on Gaze Strategies in Closed Self-paced tasks, this aspect of the driving task is discussed. It is in particular emphasized why it is important to carefully separate the visual strategies driving (quite closed and self-paced) from visual behaviors relevant to other forms of driver behavior (an open-ended menagerie of behaviors). There is always a balance to strike between ecological complexity and experimental control. One way to reconcile these demands is to look for natural, real-world tasks and behavior that are rich enough to be interesting yet sufficiently constrained and well-understood to be replicated in simulators and the lab. This *ecological approach* to driving as a model behavior and the way the connection between “lab” and “real world” can be spanned in this research is of interest to anyone keen to develop more ecologically representative designs for studying human gaze behavior.

## Introduction

Human behavior in the natural world is deeply impressive. Walking in a crowd, bicycling, or driving are carried out with an everyday ease that belies the fact they are underpinned by sophisticated cognitive mechanisms. Our capacity to encode complex 3D information, and to quickly and reliably transform it into conscious perception and coordinated action allows us to cope with the complexity and ambiguity of the world in ways that are only beginning to be understood. We are also very efficient in adapting these mechanisms to learn new skills. For example, most people learn to drive a car after only a handful of hours of driving instruction and are then ready to take their new skills to the traffic environment.

The magnitude of the information-processing challenges involved is revealed in artificial intelligence (AI) and robotics trying to develop self-driving cars. In elite performance, like motor racing, these learning mechanisms are taken to the limits of human physiological and cognitive capacity. Again, here the flexibility and efficiency of humans coping with the real world are beyond any current AI. This advantage we have over machines in dynamic tasks in the wild stands in stark contrast to tasks like chess, go and computer games (*cf*. Moravec, 1988). In traffic and sports, humans rule—in computer games and board games it is the machines that vastly outperform humans (as long as the machine is *not* required to *physically* move the pieces!). This “human advantage” suggests the human brain has discovered—in evolution and individual development—strategies and techniques for organizing perception and action that well fit our natural ecology, but which may be different from current AI. This makes dynamic, ecological tasks of particular interest to cognitive science.

Human performance in these kinds of naturalistic tasks can be studied with a number of experimental techniques. One particularly useful one is eye tracking, and analysis of gaze behavior can reveal underlying perceptual, cognitive, and motor control processes in dynamic tasks. Or, at least it can inform about the characteristics of visual input actually available to the brain, and how active gaze strategies sample, shape, and modify that information. Implications in terms of cognitive processes will require further development of rigorous, ideally computational, models of cognition in natural tasks like driving (see Lappi and Mole, 2018, for discussion).

Eye trackers can be deployed in field settings. Then, after identifying relevant aspects of the real-world task these can be recreated in simulators and lab set-ups where one can bring behavior and stimuli under more experimental control. Conversely, when lab set-ups are intended to mimic some real-world task demands, it is always an issue whether or not subjects are actually using the same strategies and skills that they would deploy in the (assumed) real-world analog… or whether the experimental task design actually allows or even encourages the use of some alternative strategy, which may be a good fit for the task but different from what is happening in the real world. With performance measures based on eye tracking (and complementary behavioral and telemetry measures), it possible to seek direct ecological validation by comparison of lab and field work.

**Figure 1:**
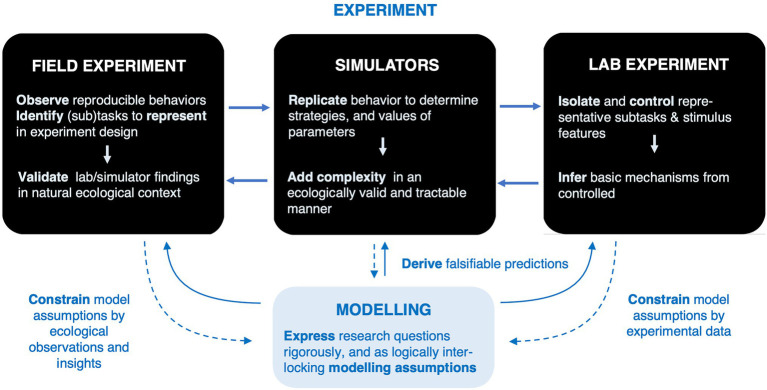
An ecological approach to natural gaze behavior. There is more than one way of making sure experiments and models capture real-world phenomena (rather than accounting for laboratory behavior only). The only way, or the best way, “into the wild” is not necessarily to take laboratory experiments and “make them more ecological,” adding more “naturalistic” features (starting from the bottom of the right-most black box). Or one may begin with real-world *observation*, that is, measurement and analysis of natural behavior not just anecdotal observation (starting in the top of the left-most box), identify candidate behaviors to experiment on and to model. Other routes are possible as well.

So, through an ecological approach of *reproducing and studying in the lab visual strategies that demonstrably occur in natural task domains, and which therefore have been adapted to deal with complexity and ambiguity of the real world (not just the experimental task)* one may hope to discover mechanisms and principles underlying “the human advantage.” One key to making such approach work is to identify natural task domains that are sufficiently constrained and stereotypical that it is possible to directly compare behavior in simplified representative tasks in the lab to real-world behaviors measured “in the wild.” As Gibson (1986, p.3) put it, *“It is not true that ‘the laboratory can never be like life’*. *The laboratory* must *be like life!”* (Figure 1).

Driving is one domain where this can be achieved:

- “The human advantage” is observed
- It is technically feasible to do eye movement research in highly naturalistic field settings with instrumented vehicles and wearable measurement systems, and
- Aspects of the real world can be reproduced with high fidelity in simulators and lab set-ups.
- What is more, many of the same methods for analyzing and interpreting gaze and telemetry signals apply across the spectrum from the field to simulators to the lab, and back.

The driving task is therefore in many ways an attractive “model system” to study humans cognition in a naturalistic ecological setting. What has been found out by such research so far?

### Aims and Structure of the Review

The purpose of this review is to cover the basics of what is known about “in the wild” human gaze behavior in driving. Its purpose is to introduce the reader to main findings on robust patterns in gaze behavior, that can be reliably identified and replicated in naturalistic task settings. Lab, field, and simulator research are reviewed, but the scope has been set to behaviors and phenomena that can be identified and quantified in the wild *and* studied in controlled settings (that is, excluding phenomena that are “lab only” or “real-world only”). Such behaviors and phenomena will be of interest for researchers working not only on driving, but in any domain involving visual guidance and control with similar task demands (e.g., many sports). Even more generally, the ecological approach and the way the connection between “lab” and “real world” can be spanned in this domain is instructive for anyone who wants to develop more ecologically representative experimental designs for studying gaze behavior.

A literature review may provide value in many ways, depending on the perspective on the literature it takes (vanWee and Banister, 2016):

- Evaluating the advantages and disadvantages of different approaches, such as lab-based vs. field approaches. This is helpful in assisting the reader to interpreting findings in the existing literature (for discussion on eye tracking methodologies from this perspective see Lappi, 2015).
- Making explicit research gaps and under-explored areas. This is helpful in guiding future research (Lappi, 2016; on driving research specifically see Lappi, 2014, 2015b).
- Proposing a conceptual model that makes explicit a theoretical structure—how existing findings and theories interlock This is useful for assessing what is currently well-understood, and what is still vague or less-than-well-founded, empirically (On driver gaze strategies in this regard, see Lappi and Mole, 2018).
- Providing an information base that is useful for a researcher coming into or returning to a field, who wants a primer or refresher of the state of the art. This is the purpose of the present review. (For a broader overview of human gaze behavior across naturalistic tasks, Land (2006, 2019) are excellent reviews).

The literature reviewed here will provide core readings for researchers working on oculomotor behaviors and physiology in the lab who wish to extend their research into driving, or other naturalistic visual-locomotor tasks, or on the other hand researchers in more applied fields (sports, transportation) who are interested in bringing aspects of the real-world ecology under experimental scrutiny.

The paper is written in a way that would be accessible and interesting to non-specialist researchers (i.e., oculomotor researchers not doing research on driving, and/or driving or human movement researchers not doing research on eye movements)—while remaining of interest to practitioners and domain experts, and without oversimplifying the ecological complexity of real-world visual behavior.

The rest of the paper is structured as follows: “Why Study Driving?” outlines the deeper theoretical motivation for studying driving, and the characteristics of driving that make it an attractive model system to understand human behavior “in the wild.” “Driving as a Closed Self-Paced Task” describes the key properties of the driving task. Part of a Research Topic on Gaze Strategies in *Closed Self-paced tasks*, this aspect of the driver’s task is emphasized. Here, it is important to carefully analyze the *driving* task itself and separate it from other forms of driver behavior. “Primary Gaze Strategies” reviews empirical work on gaze strategies in driving. “Discussion” closes with discussion of some of the theoretical implications and open issues.

## Why Study Driving?

Since Gibson and Crooks (1938), driving has been an empirical testbed for understanding the visual control of locomotion in complex 3D environments. In terms of visual guidance and control, it is one of the best understood forms of human locomotion. This is partly thanks to the amount of experimental work with field set-ups, driving simulators, and simplified steering tasks in the laboratory have yielded detailed observations in experimental psychology. What is more, cognitive modeling work can also leverage knowledge gained in vehicle engineering and autonomous driving, such as the relevant dynamics and feedback control principles.

There are a number of characteristics that make driving an attractive model behavior to study (summarized in Table 1):

1. Ecological validity.
  1.1. Field experiments are feasible. Driving behavior can be investigated in real vehicles, on the road, and track. This of course yields maximal ecological validity in terms of the sensory information sources and consequences of motor actions.
  1.2. It is possible to do experiments across the full spectrum: from measuring fully naturalistic behavior in the field, through high-, medium-, and low-fidelity simulations, to simplified and restricted (but still representative) laboratory tasks, and to balance ecological complexity against experimental control in the experimental set-up, analysis pipeline and choice of physiological measures and performance metrics.
  1.3. Besides the attraction of field studies and high-fidelity simulators, there is another, subtler aspect to ecological validity of simulator laboratory tasks. As a real-world skill, driving is something people already do outside the laboratory. So, driving tasks in “measurement and modeling friendly” lab tasks can still tap into skills and knowledge that has adapted, over a period of time, to the “measurement and modeling unfriendly” real world. One can readily find experienced test participants who have developed skills and strategies that they use for coping “in the wild” and bring them to the lab to study. (This is in contrast to participants having to familiarize themselves with and learn an artificial task, following explicit instruction). This can help make the laboratory, and behavior observed in it, more “lifelike.”
2. Empirically demonstrable representativeness. This lifelikeness, moreover, need not rely on merely intuitive judgment or face validity. Lab/sim results in reproduced driving tasks can be directly compared to real-world physiological measures and performance. That is, it quite often makes sense to measure and model the very same things both in the lab and in the wild allowing direct and quantitative assessment of external validity (one key contribution of this paper is to show examples of this, that is, focus is on behaviors and measures that can be observed across the full ecological spectrum).
3. The whole spectrum of skill development also can be covered. The fundamental task driving along a road is meaningful both to the complete novice (driving school student) and to world class elite (champion racing driver). In many fields of expertise and expert performance, by contrast, a task that presents a meaningful challenge to the expert and allows differentiation at the highest levels of skill can be impossible for the novice to perform, and a task that is doable for the novice can be trivially easy for experts.
4. Simple 3D scene layouts. The driving environment is engineered to have a fairly simple and stereotypical 3D layout (as opposed to the ruggedness and clutter in most natural and built environments). This makes the driving task more amenable to computational modeling than behavior in more cluttered and unpredictable environments. (Some consequences of this point are expanded on in the next section).
5. Convenience of instrumentation.
  1. 5.1. In fieldwork, it is in practice physically easier to instrument a car with, say, eye trackers than to develop wearable instrumentation. Also, positional and dynamic data can be recovered from GPS, dataloggers and vehicle CAN bus.
  2. 5.2. High-grade purpose-built equipment is commercially available—to do lab, simulator, *or* field experiments.
  3. 5.3. Procedures for data logging, signal analysis, and interpretation in terms of driver/vehicle performance are well established and understood.
6. Low dimensional controls. Compared to many naturalistic dynamic tasks, driving is quite significantly constrained. In driving, locomotor control is achieved through the steering wheel and two pedals. All the skill and experience of a driver must ultimately be expressed through these quite simple controls, with just three degrees of freedom. (This is arguably an orders of magnitude reduction in complexity, compared to modeling, at comparable level of detail, say, visual guidance in downhill skiing where the biomechanics of the muscles limbs and joints wound need to be modeled). Again, this simplifies analysis of performance and allows meaningful comparison of experts and novices.
7. A hundred years of research on driving in psychology and engineering gives a solid base to work from.

**Table 1:** From an experimental and modeling point of view, gaze strategies in driving are a particularly attractive “model behavior” for understanding visual guidance in ecologically representative tasks.

1. Ecological validity. 1.1. Field experiments are feasible 1.2 The whole spectrum from field studies through high-fidelity simulators to restricted laboratory designs can be covered. 1.3 Participants adapted to “natural” task demands over a long period of time readily available.
2. Empirically demonstrable representativeness.
3. The whole spectrum of skill development can be covered (novice to elite level experts).
4. Simple 3D scene layouts.
5. Instrumentation. 5.1. Physical convenience. 5.2. Availability of high-grade equipment. 5.3. Established methods of data collection, analysis, and interpretation.
6. Low dimensional controls.
7. A hundred years of research on driving in psychology and engineering gives a solid base to work from.

The need to understand the driver–vehicle system dynamics in engineering has led to the development of mature driver-in-the-loop simulations that psychological and cognitive modeling work can be leveraged on. The past 25 years with mobile eye tracking has amassed a substantial amount of observations and data on natural gaze behavior. The current technological push to develop machine vision and machine learning systems for autonomous cars makes the issues timely and offers opportunities of cross-fertilization between cognitive science and AI.

## Driving as a Closed Self-Paced Task

The road environment is a fixed, stable 3D layout with well-understood parametrization because it has been engineered to allow humans to safely and comfortably travel at high speeds. A perhaps non-obvious implication of this, as far as task demands are concerned, is worth pointing out. Although one sometimes hears that we “did not evolve for” the speeds modern vehicles easily achieve, this statement somewhat misses the relationship between speed and distance in determining the *rate of events* that need to be processed (Senders et al., 1967). The engineering of the everyday modern road environment is such that higher speeds are only achieved on very open roads, with large curvature radii and little visual clutter. On twistier roads, or in more cluttered environments, the speeds are lower. And, critically, speed selection is in control of the human driver: the driver actively *adjusts* speed to maintain task demand (the rate of information processing required) at some desired level (Taylor, 1964; Senders et al., 1967; Näätänen and Summala, 1976; Fuller, 2011). This makes driving a se*lf-paced task*.

The simplicity of the 3D scene layouts, and the constraints on control actions make driving a more *closed* task than many real-world skills, which is a distinct advantage for ecological researchers looking for “model behaviors” in the wild that can be studied and brought to the lab. The aim of ecological design in lab tasks is to make them simplified but representative. Lab settings are necessarily simplified, and the more simple naturalistic setting one is aiming to capture, the more representative it will be. Thus, researchers should be on the look-out for *closed* naturally occurring task ecologies.

### Driving vs. Driver Behavior

Note that the above applies to *driving* specifically—not to “driver behavior” generally, which is defined here as the totality of everything people do while in control of a vehicle. This paper focuses on driving. There is a fundamental theoretical reason for this. Driver behavior is a menagerie of behaviors that has emerged at a specific point in history and varies across different cultures. It may have applied interest—for example, in traffic safety research or human factors engineering, perhaps cultural history interest as well—but lacks deeper scientific interest. For basic science, there is interest in driving, however, because of what it can reveal about the perceptual-cognitive strategies of visually guided steering, speed control, and trajectory planning. For this purpose, it is indeed an excellent model system for investigating the mechanisms and strategies humans use—in natural activities they actually do also when they venture outside of the lab.

This distinction between driving and driver behavior has some practical implications for the analysis of eye tracking data as well. For in real-world driving, the relevant gaze strategies are embedded among visual strategies serving other driver behaviors: any observed strategies serving the driving task “in the wild” will be *interleaved* with gaze behavior relevant for other tasks.

This will occur the more so the more the driver is multitasking other non-driving behaviors, especially ones that need foveal vision or visual tracking as a resource. For example, operating a sat nav or adjusting the radio will require the driver to avert gaze from the road environment, to a visual display and/or touch buttons. This is of course why Human–Machine Interface engineers design steering wheel controls and voice command control into the interfaces of such non-driving-related devices. Even tasks that do not themselves use gaze as a resource may still interfere with motor planning or task-switching that is needed to juggle between subtasks. Thus, even if the secondary task—like, for example, doing mental arithmetic of having a conversation—has no visual component, it may introduce a “cognitive load” significant enough to interfere with the time-sharing mechanisms needed for switching between tasks (or between subtasks within the driving task). In this case, the measured gaze behavior may exhibit a reduction of saccadic scanning (Lehtonen et al., 2012).

**Figure 2:**
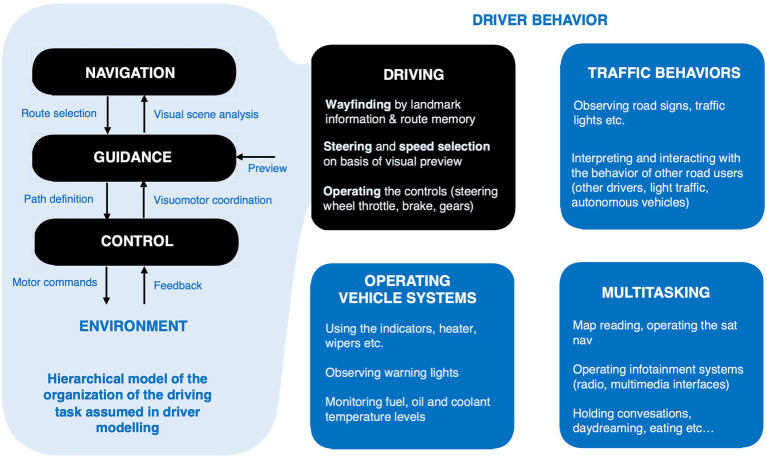
Task structure of driving vs. driver behavior. While the driving task can be described in terms of steering and speed control and visual anticipation with gaze, it does not follow that the underlying brain processes compose in this way (e.g., separate steering and speed control “systems” or “modules,” or gaze strategies specific to steering and gaze strategies specific for speed control).

What follows will focus on what we know about gaze strategies in *driving*, as opposed to driver behavior in general (Figure 2). Here the most detailed work has been on *visual guidance*. It is the core skill in driving, and indeed any high-speed locomotion, and a process where more insight can be gained by leveraging psychological theory on the parallel and complementary work on feedback and feedforward control in systems engineering (for a review and historical comparison of the psychological and engineering approaches see Lappi and Mole, 2018). This is a level of organization within the driving task that is intermediate between the choice of route from among alternatives (navigation) and the actual motor coordination needed to follow the desired path (control). It relies heavily on *visual preview*, which is actively sampled with eye movements.

## Primary Gaze Strategies

Even under our “narrow” definition, driving is a complex task. It involves perceptual processes for *multisensory integration of sensory signals* (visual, auditory, vestibular, somatosensory, and proprioceptive). Signals from the same event are encoded in *multiple coordinate systems* (sensor level, egocentric, allocentric), and arrive at different latencies. Motor programs must integrate complex and precisely timed action sequences across multiple effectors (eye-hand-foot coordination). At the cognitive level, the stimulus information available for a driver is rich, complex, and noisy and must be combined with vast amounts of information from experience, which is stored in memory. All this must happen under severe time constraints (but, as said, how tight these constraints are in any given situation is in part a tactical decision of the driver, exposure to different task demands being regulated *via* speed control).

Thus, while driving is a closed-enough task to be approached with computational modeling and AI, it is also open enough to be of interest for the problem of the human advantage. In terms of gaze behavior, it exhibits all the “laws” of gaze control in the wild (Table 2). What are these “laws”?

**Table 2:** Seven recurring “laws” of gaze behavior in the wild (Lappi, 2016) are exemplified in driving.

**1. Repeatable and stereotypical gaze patterns across subjects** 1.1. Fixation-saccade-fixation sequences, which are typical of almost all natural tasks (although what counts as a “fixation” in a natural task is itself a non-trivial question Lappi, 2016; Hessels et al., 2018). 1.2. Gaze is mostly concentrated in the Guiding Fixation region (far road), with a pattern of optokinetic nystagmus (Authié and Mestre, 2012; Lappi et al., 2013; Lappi and Lehtonen, 2013; Itkonen et al., 2015), with occasional fixations of the “tangent point” at the curve apex, especially during curve approach and turn-in (Land and Lee, 1994). Guiding fixations have a time headway of about 2 s (Lappi and Lehtonen, 2013; Lehtonen et al., 2014; Tuhkanen et al., 2019). 1.3. Saccades are used to scan further (look-ahead fixations, forward polling; Lehtonen et al., 2013, 2014), back to the near road (return fixations, backwards polling; Navarro et al., 2021) as well as at the scenery, mirrors, and instruments. 1.4. Experienced drivers in traffic generally have broader scanning patterns than novice drivers (Crundall and Underwood, 1998). Much of this will be scanning the traffic environment (intersections, traffic, etc., *cf*. Lemonnier et al., 2020), but part of this may be a difference also in how *far* experienced scan with their look-ahead fixations (Lehtonen et al., 2014). **Note:** More detail on stereotypical patterns in the text.
**2. Gaze is highly focused on task-relevant targets** 2.1. In curve driving, the guiding fixations occur in small visual region on road ahead (not more than 10° horizontally, 5° vertically; e.g., Land and Lee, 1994; Itkonen et al., 2015). This region acts as a visual pivot for scene exploration and analysis. 2.2. Other targets must be identified very precisely in the periphery as they are fixated with precise saccades (*cf*. Land et al., 1999; Lappi et al., 2017;). **Note:** 2.1. indicates top-down control based on interpretation of the situation (understanding 3D layout of the scene, situational awareness of multiple targets and obstacles) rather than the visually most salient ones repeatedly “capturing” gaze. 2.2 Indicates that peripheral vision is much more useful than many textbook accounts of (based on retinal acuity and cortical magnification considerations) might lead you to believe (Wolfe et al., 2017).
**3. Function of even individual fixations can be interpreted (though not always in an *a priori* transparent or intuitive way)** 3.1. Guiding fixations can be interpreted as fixation of different *steering points* proposed by various steering models (e.g., Land and Lee, 1994; Land, 1998; Wann and Swapp, 2000; Salvucci and Gray, 2004; Wilkie et al., 2008; Boer, 2016; for reviews see Wann and Land, 2000; Lappi, 2014). 3.2. Fixations at different depth distances and even in the scenery could maintain a “spatial image” of the scene layout (see note in L6, below). 3.3. Relevant targets, such as other road users, are also “monitored.” **Note:** The measured patterns of gaze, while repeatable within and across individuals, can be surprising to the subject (eye movement control is highly implicit) and even the researcher. The details of how the spatial image is monitored and how multiple targets are monitored and selected need ongoing work. Proper interpretation requires rigorous theories of the underlying perceptual-cognitive processes that the eye movements serve.
**4. Targets tend to be fixated “just in time”** 4.1. Unless a (sub)task requires continuous monitoring/tracking, gaze disengages from the previous target and switches to the next just before (sub)task completion. That is, each target is fixated right at the moment they become relevant for guiding the next action (Ballard et al., 1995). In many natural tasks gaze tends to lead action with about 1 s lead time. This is also the case with the coupling of driving and steering (Land, 1992; Chattington et al., 2007), or glancing the mirror before lane change etc. **Note:** Scanning early would require maintaining the gleaned information in short-term memory, requiring more cognitive resources, and running risk of obsolescence of that memory. Instead, humans generally prefer use a “do it where (and when) I look” strategy.
**5. Visual sampling is intermittent** 5.1. Gaze is highly labile. There are no fixation crosses in nature. We do not “stare” at a single target for a prolonged period of time. The visual world is sampled with 2–4 fixations per second, interspersed by saccadic suppression. 5.2. Blinks (0.1–0.3 s duration) also break up the visual input. Expert racing drivers that are highly reliant on high-quality input seem to tactically perform blinks at specific, less critical parts of the track (Nishizono et al., 2021). 5.3. “Just-in-time fixations” (or guiding fixations, GF) are interleaved with look-ahead fixations (LAF) in driving, this happens, for example, in approaching a bend (Lehtonen et al., 2012, 2013, 2014; Schnebelen et al., 2019; Navarro et al., 2021). 5.4. In multitasking, fixations for one task are interleaved with fixations to targets relevant to a parallel task. Gaze time is shared between tasks. For example, glancing at the instruments can be interpreted as a form of intermittency (Johnson et al., 2014).
**6. Humans remain oriented to, and orient gaze in relation to, targets currently outside the field of view** Memory contribution to driving has been little studied, but Shinoda et al. (2001) show that traffic sign detection in expected locations is better than for signs in unexpected locations. Expert racing drivers rely on a rich memory representation of the track (Lappi, 2018). Land and Tatler (2001) studied their visual strategy, and orienting gaze, head and the vehicle seems to account for road geometry at ranges beyond the current view (*cf*. also vanLeeuwen et al., 2017). **Note:** In itself the fact that there are *few* fixations to irrelevant objects (2.1. and the precision of saccades 2.2.) implies knowledge of where the task-relevant objects and locations are in 3D space (*cf*. e.g., Land et al., 1999). This implies trans-saccadic memory (Tatler and Land, 2011) or an “image” of 3D space (Loomis et al., 2013; *cf*. Senders et al., 1967; “expectancy” in Näätänen and Summala, 1976). Interestingly, this “image” does not seem to be able to support simple and over-learned sequential steering manoeuvres in the absence of feedback (Wallis et al., 2002, 2007; Cloete and Wallis, 2009). Investigating the nature of the “spatial image” in driving could benefit greatly from the rapidly advancing literature on scene representation and wayfinding in cognitive neuroscience (Barry and Burgess, 2014; Epstein and Vass, 2014; Spiers and Barry, 2015; Epstein et al., 2017; Patai and Spiers, 2021).
**7. Gaze control is “embodied”: oculomotor control is embedded in integrated eye/head/body/ locomotor control** 7.1. Control of the *gaze* is not achieved only by rotation of the eyes (i.e., control of *oculomotor events*), but also by rotating and translating the head and the body. 7.2. Conversely, head and body movements are compensated by gaze-stabilizing eye and head rotations. **Note:** This suggests that gaze control is not a “modular” problem, and how gaze strategies emerge in the wild may not be decomposable into separate contributions from the traditional “oculomotor systems,” as isolated and studied in lab (discussion of this theoretical position can be found in Steinman, 1986 and Lappi, 2016).

For more detail and empirical examples, see Lappi et al. (2017).

Eye tracking in naturalistic tasks, outside the confines of typical laboratory behavior, has begun to reveal consistent patterns of gaze behavior that tend to remarkably regular and repeatable within and across participants doing the same task. Gaze patterns and visual strategies in numerous tasks, such as making tea (Land et al., 1999), sandwiches (Hayhoe et al., 2003), foot placement in rugged terrain (Matthis et al., 2018) have been studied, steering a car (Land, 1992; Land and Lee, 1994; Lappi et al., 2013, 2017, 2020) and sports, such as batting in cricket (Land and McLeod, 2000; Mann et al., 2013) and squash (Hayhoe et al., 2012).

This work, which has begun to reveal commonalities in the patterns of gaze behavior in natural tasks, is reviewed in Regan and Gray (2000), Hayhoe and Ballard (2005), Land (2006), Kowler (2011), Tatler and Land (2011), Tatler et al. (2011), Lappi, 2016, Hayhoe (2017). Lappi (2016) proposed that many of the common findings can be summarized in terms of seven “qualitative laws” of gaze behavior in the wild: reliably recurring patterns (across different tasks) that tend to go together, the more so the more naturalistic the setting—all of them expected in extended sequences of fully naturalistic gaze behavior. Lappi et al. (2017) demonstrated, that all of them indeed do go together in a fully naturalistic sequence, *viz*. an experienced driver along a (rural) road. This indicates that gaze behavior can be usefully employed as a model behavior representing many of the general findings in the literature on natural tasks. It is to the detailed investigation of these behaviors that we turn to next.

### Eyes on the Road

**Figure 3:**
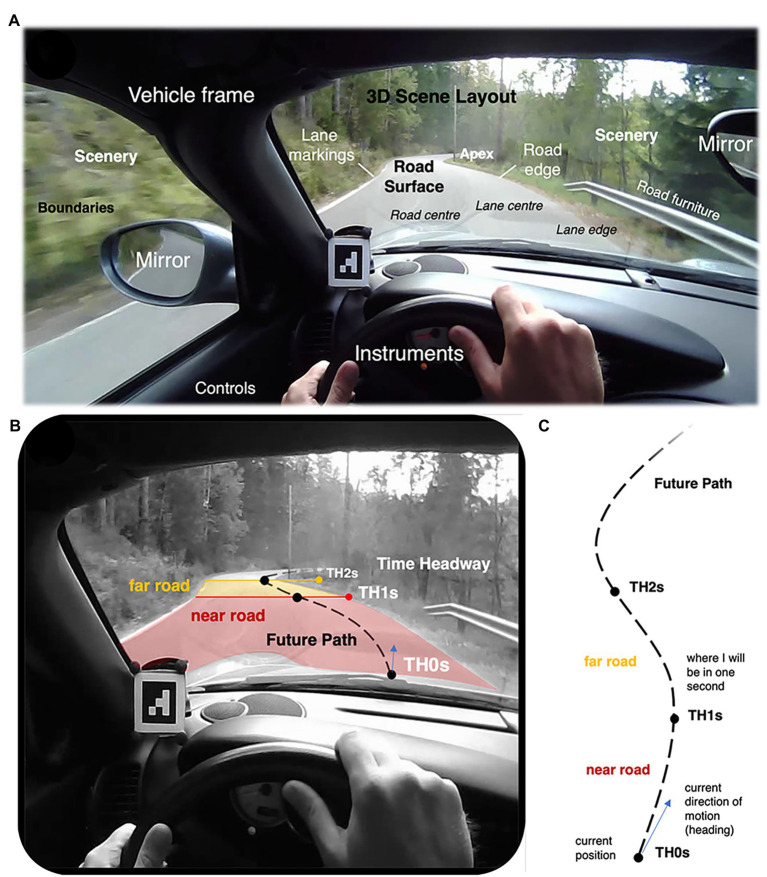
**(A)** Field of view of the driver. **(B)** Parsing “the road” into future path, and near and far road, based on time headways. The underlying image is from the supplementary movie in Lappi et al. (2017), from which the reader can get a feel for the dynamic behavior. **(C)**. Bird’s eye view of the future path and time headways.

A driver’s field of view (Figure 3A) contains the 3D scene flowing by (this is apparent motion within the field of view caused by self-motion through a fixed 3D layout—optic flow, Gibson, 1958, 1986; Cutting, 1986). The view of the road scene is bounded by the vehicle frame. The visual preview of the road scene is the perceptual source of anticipatory information for steering and speed selection; vehicle instruments and mirrors provide additional information about the surrounding 3D scene (the view behind), about one’s movement through it (speedometer), and about vehicle dynamic state (RPM gauge). An experienced driver does not need to avert eyes from the road to operate the vehicle (gearshift, indicators), but some controls, such as dipped beams, may require averting gaze from the road scene. In familiar surroundings, anticipatory information is also available top-down, from route memory. Feedback information, including non-visual feedback, for example, of vehicle stability is also important for steering and speed.

The physical scene itself is a stable 3D layout, defining boundaries within which the road surface is embedded. The scale, curvature, and grip of the road surface are main determinants of appropriate speed selection, and thereby of the pace and difficulty of the task (rate of events Senders et al., 1967; Kujala et al., 2016; task demand Fuller, 2005, 2011). The road surface is usually smoother and flatter than the more rugged scenery surrounding it. It is sometimes, but not always, indicated by markings for road or lane edges. Apices of curves ahead provide powerful a visual cue of upcoming bends, and the imminent steering requirement. These are fundamental properties of the driving task, independent of the kind of vehicle one is using (indeed, they hold whether or not one is using a vehicle at all).

Road furniture, such as railings, lamp posts, trees, buildings, and traffic signs, add visual clutter and give additional constraints to speed selection (such as limited sight lines or posted speed limits). They also give clues about the traffic environment (e.g., rural vs. urban area tend to have different traffic—*cf*. the “self-explaining road” idea, Theeuwes and Godthelp, 1995). Other road users are also embedded in this scene (i.e., constrained to move on the ground). Traffic laws and informal social norms for “rules of the road” add another semantic layer of complexity to the interaction between the driver and other road users. Passengers and in-vehicle navigation and infotainment systems may bring into driver behavior still further tasks (map-reading, conversation, singing etc.) that are multitasked with driving. These aspects are more specific to the modern traffic environment and vehicles, and/or relate to other driver behaviors beyond driving.

Where do drivers look? It is generally accepted common sense that drivers should “keep their eyes on the road.” But for a scientific understanding one must move beyond this, to ask: *where* and *when* is gaze deployed, in order to glean *what* information, for the use of *which* perceptual-cognitive and motor control processes?

And somewhat more subtle, but methodologically fundamental, is the question of how gaze targets in the world should be classified and quantified in the first place. Natural task environments do not come ready-parsed into *a priori* defined “stimuli.” The road scene is not a sparse collection of objects floating on the (wind)screen, and unambiguously distinguishable by a few visual features. Instead, it is a rich array of task-relevant objects and locations, arranged into a scene. And natural task demands that determine *which* objects and locations are task-relevant are not completely known *a priori* to the experimenter. (In an ecological task what is “task relevant” does not follow from the experimenter’s task instruction, but actual ecological relevance for the subject). One of the key questions in analyzing real-world data—or making decisions on what features to reproduce, or simulate with what fidelity—is to first determine what would be an *ecologically meaningful* representation of the task environment. So, perhaps paradoxically, before one can ask *how* drivers keep their eyes on the road, by what mechanisms, one must first define *what* “a road” *is*. (i.e., what are the appropriate scientific concepts to describe the most relevant aspects of the road environments).

As a time-constrained task—or more precisely: a self-paced task under fixed constraints from scene layout and mechanical and dynamic constraints of body/vehicle—it is often useful to think of the driving task in terms of *time margins* (Lee, 1976; Godthelp et al., 1984; Godthelp, 1986; see also Lee et al., 2009). These can be for example time headway to a leading vehicle, time-to-contact to an obstacle, time-to-line crossing in curve negotiation etc. Perhaps the most fundamental and general ones for analyzing steering, speed selection (and, as we shall see, gaze strategies) are the *time headways* to different points on the future path: the amount of time it would take to arrive at a location on road and/or draw parallel to an object on the side of the path. These time headways can be used to define a “near road” region (under 1 s headway) and “far road” region (about 1–2 s time headway; Figures 3B,C).

For the researcher, these time margins give a convenient framework wherein gaze strategies can be economically represented and usefully interpreted. In ecological experiment design and/or the analysis of real-world data, it is often a crucial consideration how the data should be parametrized, and what coordinate systems signals should be projected to (on these issues with regard to eye movement research see Lappi, 2015, 2016; Hessels et al., 2018). If there is already a well-established way to parametrize task environment and dynamics in a domain this offers the researcher a powerful head start in modeling and interpretation (see also section “What Do Guiding Fixations Look Like?”.).

For the driver, these time margins are psychologically important as *safety margins* (Summala, 2007)—also a critical consideration given inherent physiological constraints in how fast perceptual information can be transmitted into the brain, the time cost of analyzing it, and the motor delays in issuing responses. Some of these constraints come directly from the design of the visual system. There are limits to how fast we can move the eyes, and how long fixations in between eye movements are needed to transmit reliable signals into the brain. Also, saccadic suppression and masking (Thiele et al., 2002; Wurtz, 2008; Ibbotson and Krekelberg, 2011) will place limits on the rate of saccades one can make while maintaining useful vision. Cognitive processing and task-switching costs in multitasking will produce additional delays.

### Guiding Fixations as a Visual Pivot

**Figure 4:**
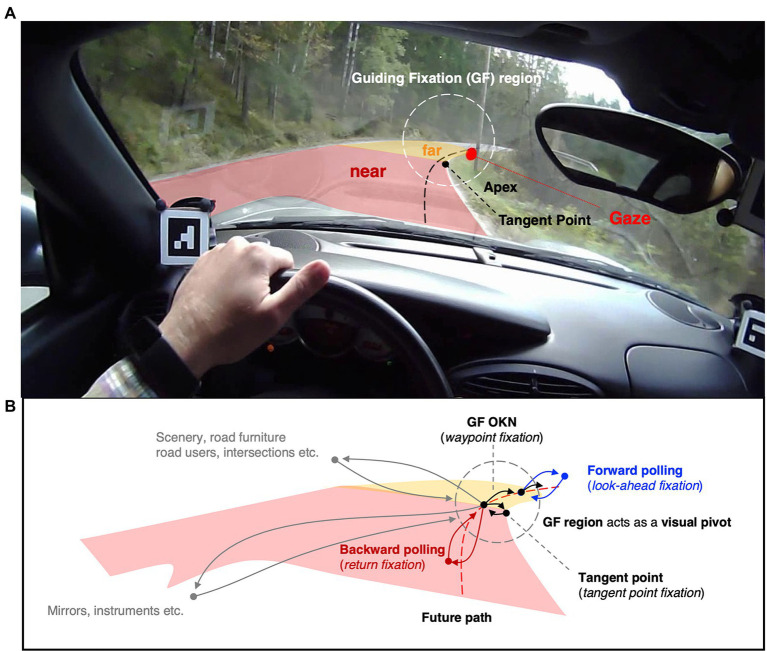
Guiding fixations and the visual pivot strategy. This frame in this is taken about 2 s after the frame in Figure 3: the driver has now arrived at the far road (TH2s) region and begun to turn into the bend. Gaze is looking ahead, guiding steering, and anticipating the upcoming end of the bend. **(A)**. Most of the time gaze is concentrated in a small region of the visual field—the guiding fixation (GF) region. The concept of guiding fixations can be operationally defined in terms of time headways, between about 1–2 s TH. **(B)**. The GF region acts as a visual pivot, from which saccades are launched, and to which saccades return. Scenery and in-car fixations are eyes off the road fixations, the rest are eyes-on-the-road fixations. Gaze polling: i. saccade lands further ahead than the far road GF region and returns (look-ahead fixation, forward polling) ii. saccade made back to the near road and the back to GF region (return fixation, backwards polling). Tangent point fixation: saccade to the tangent point (a travel point on the inside lane edge). GF OKN: future path waypoint (or reference point) fixation. For these Guiding Fixations (the majority), the eye does not remain stable in its orbit: these guiding fixations are “tracking fixations.” The line of sight is locked onto locations in the 3D scene one is moving through. (Note: This is a schematic representation; what is here indicated by individual gaze points may be glances comprised of multiple fixations; and not all glances in the periphery always return to the pivot in a rigidly mechanical way).

Experiment has shown that drivers’ gaze tends to be highly concentrated in the *far road* region (TH 1–2 s). This is true for both real and simulated driving, and modeling indicates that the far road is the region where the most path information for steering can be gleaned (for discussion of the history of different models in this context see Lappi and Mole, 2018). Therefore, fixations to on-road targets in the far region of the visual field are often referred to as *guiding fixations* (Figure 4A).

A terminological point, wherein research on oculomotor fixations in typical lab experiments and gaze fixations in naturalistic tasks differ: that “fixations” (including most guiding fixations) are often fixations of a fixed object or location. That is, they do not look like oculomotor fixations where the eye is fixed in the socket, if the subject is moving relative to said object. Therefore, the concept of “fixation” itself can differ between studies (see Lappi, 2015, 2016, and Hessels et al., 2018 for discussion).

What is the role of these guiding fixations? A preliminary word of caution on theoretical interpretation is in order. The cognitive function of a fixation cannot be read off from the *gaze position* in the visual field—calling far road fixations guiding fixations is therefore a functional interpretation. Further, determining which of the many theoretically possible targets within the guiding fixation region (if any) the driver is actually looking at is not a trivial question: because of the geometry of visual projection, all targets in the guiding fixation region are projected very close to each other into the visual field, meaning it is not feasible to differentiate between them based only on where the measured gaze position is. (Even with very good signal accuracy and calibration, reasonably sized areas of interest (AOIs) placed at the targets inevitably overlap; for more detailed methodological and theoretical discussion see Lappi (2014)).

Guiding fixations are interspersed by occasional *gaze polling* (*cf*. Wilkie et al., 2008): *forward polling* or *look-ahead fixations* to the road beyond the 1–2 s time headway (TH) far road region (Lehtonen et al., 2013, 2014; Schnebelen et al., 2019) and *backwards polling* for *return fixations* to the *near road* region (Navarro et al., 2021). Guiding fixations and gaze polling characterize the overall pattern of “eyes-on-the-road” fixations. They are interspersed by “eyes off the road” glances to the instruments, mirrors, scenery etc. When other road users are present they are of course frequently fixated and sometimes tracked. Overall, driver eye movement patterns seem to form what Vater et al. (2020) call a *visual pivot* strategy. This type of strategy is used when there are multiple task-relevant objects or locations in the scene that need to be fixated (are too far apart to be visually resolved simultaneously): gaze shifts from one target to another in sequence, the saccades launching from and returning to the pivot. In driving, the guiding fixation region forming the pivot, and gaze polling and off-the-road glances the spokes (Figure 4B).

While guiding fixations are tightly coupled to steering (with about 1 s *lead time* between gaze rotation and body/car rotation—note that lead time and time headway are separate concepts), the look-ahead fixations are not. Look-ahead fixations are used in many tasks to glance targets that will become relevant for later actions (Ballard et al., 1995; Pelz and Canosa, 2001; Mennie et al. 2007). In driving, it is natural to interpret this in terms of trajectory planning (Wilkie et al., 2008; Lehtonen et al., 2013, 2014). At least on public roads, the anticipation of oncoming traffic is another a major reason to scan the road ahead, especially in blind bends. But the fact that this behavior occurs in open, well-sighted turns as well (Lehtonen et al., 2014) would suggest they also serve a role in supporting trajectory planning or higher-level, sequential motor planning (for more technical discussion see Lappi and Mole, 2018).

What has been said above of guiding fixations and visual pivot strategy hold for everyday driving (only). There is very little work done on expert racing drivers (Land and Tatler, 2001; van Leeuwen et al., 2017; Lima et al., 2020) but what evidence there is and anecdotal material outside the scientific copy of record^1^ indicates that racing drivers use a “visual anchor” strategy instead: the guiding fixation (very far up the track) is very stable, and the saccades characteristic of a visual pivot strategy are absent. Instead, the 3D layout and scenery is observed peripherally, with occasional glances at the instruments and control knobs on the steering wheel. Compared to everyday driving, expert driving skill remains largely unexplored, however.

### What Do Guiding Fixations Look Like?

Detailed investigation of the gaze patterns within the guiding fixation (GF) region has yielded additional information about guiding fixations, and clues to the perceptual-cognitive processes they serve. The patterns are best understood when the gaze behavior is looked at in time series form. There is then a very robust and characteristic “sawtooth pattern” of optokinetic nystagmus (OKN; Authié and Mestre, 2012; Lappi et al., 2013, 2020, Figure 5). This is particularly well-defined and easy to observe in the horizontal gaze position signal (temporonasal component of EOG) when turning into, and cornering in bends.

**Figure 5:**
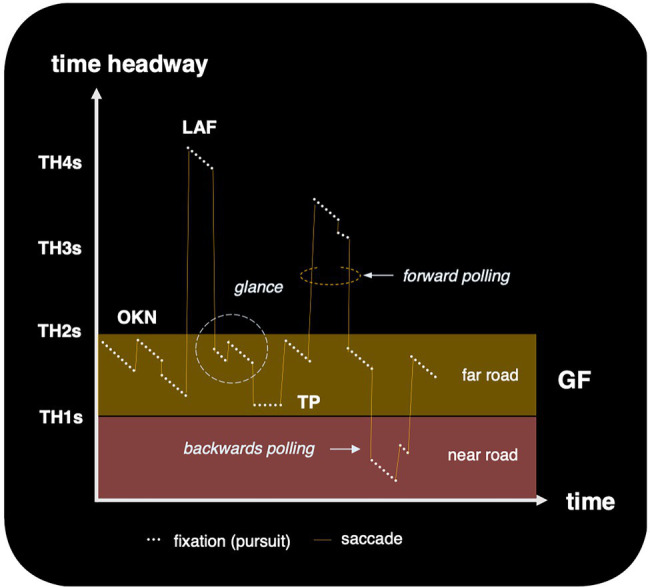
Schematic outline of the primary eyes-on-the road gaze strategies. When fixating a waypoint location the time headway reduces as one approaches it; the “fixation” is an oculomotor pursuit (when fixating *any* fixed reference point in the 3D scene one is moving relative to, the fixation is a pursuit). When fixating a travel point the time headway is constant. Also note that a “fixations” reported in natural tasks are often “glances” (i.e., the criterion of how large a saccade must be to “break fixation” can different in naturalistic and oculomotor research). TH, time headway to the point of fixation; OKN, optokinetic nystagmus; TP, tangent point; GF, guiding fixations; LAF, look-ahead fixations.

Herein lies an important methodological point: this pattern *only* becomes pronounced when the gaze position is plotted as a time series. It can be easily missed if simply looking at gaze overlay videos or gaze position aggregated over time. Indeed, some earlier field experiment papers explicitly claimed that they do not observe tracking fixations their data (Kandil et al., 2010—but see Lappi et al., 2013 for an essentially identical experiment and unambiguous OKN). Marple-Horvat et al. (2005) and Wilson et al. (2007) also report a negative finding, though here the reason may be that they used a rally game and the instruction was to drive as fast as possible, which might elicit a different gaze strategy—*cf*. the discussion of visual pivot vs. visual anchor, above. Another reason might be how well moving texture was replicated in their fixed-platform simulation set-up. This highlights the importance of the choice of frame of reference one uses to visualize and/or analyze one’s data in, which is particularly important when working with data collected “in the wild” (for methodological discussion see; Lappi, 2016), and the importance of considering the task analysis of one’s simulated task as well as the question of what stimulus features are crucial to reproduce with high fidelity (for methodological discussion see Lappi, 2015).

The functional meaning of this OKN pattern becomes clearer if one considers it in terms of where the gaze lands in the 3D scene with different types of gaze strategies (i.e., where the point of fixation is in the physical world), and how this location changes over time. Stable gaze in the visual field would imply that, as the line of sight moves with the observer, the point of fixation in the world would travel ahead of the observer. The driver would be looking at a *travel point* at a constant distance ahead of the car that is pursued but never intercepted. Optokinetic gaze, on the other hand, where the point of gaze in the visual field follows the pattern of optic flow generated by observer motion, means that the point of fixation remains fixed at a stable spot in the 3D scene: a visual reference like an object on the roadside, a crack in the asphalt, or a *waypoint* on the road one is moving toward. Looked at from the oculomotor control perspective: the slow phase of OKN is a “tracking fixation,” visual pursuit of a target moving relative to the observer (but here the relative motion is caused by observer motion).

This has implications for the analysis of the visual guidance of steering. If we think of the guiding fixations as providing visual input to steering control, then the type of information provided by the two strategies would be quite different. First, a travel point fixation will keep gaze locked onto a steering point that moves in the world ahead of the observer (e.g., a travel point on the future path at a constant TH, or, in curves, the tangent point). A waypoint fixation on the other hand locks target onto an object or a location one is going to *intercept*: the time headway to the point of gaze is not constant but rather comes down as one moves toward the fixed waypoint. Second, the travel point can in principle be fixated for arbitrarily long periods of time (it is always “there,” ahead of the observer) whereas a waypoint can be fixated for only a short amount of time, because it disappears from view as one intercepts it (and one has to switch to a new waypoint, though in practice the saccades to the next waypoint occur much earlier than interception of the previous one, see, e.g., supplementary movie 1 in Tuhkanen et al., 2021). This means that with a waypoint fixation strategy, the visual input is necessarily intermittent snapshots, interspersed by saccades, rather than a continuous stream. Eye tracking is the (only) means to determine the properties of this input in natural behavior. How the brain copes with—or perhaps takes advantage of—this intermittency in visual guidance remains an open question, for future computational cognitive modeling to address.

## Discussion

Advances in mobile eye tracking, research in increasingly realistic simulators, and increasing modeling sophistication in computational techniques have over the past 50 years built toward an increasingly detailed understanding of human gaze strategies in complex naturalistic task settings (including driving). The challenge of developing machine vision and machine learning systems for mobile robotics (including autonomous driving) make the domain pertinent and timely. Understanding how humans and other animals cope with real-world task demands is one key component in figuring out “the human advantage” we still hold over machines in natural task domains. Theories of cognitive processes involved must be grounded on behavioral and physiological data collected in rigorous and representative experiments.

In the search for ecological validity in experimental work, it is of course desirable to be able to do precise measurement and modeling in fully naturalistic task environments. An ecological approach, however, does not equate “experimental work in the real world only.” Rather, it means that lab work, which is indispensable because of the experimental control and analytic rigor it affords, must be relatable to behavior in the real world—and demonstrably so: in terms of being able to show that the same behaviors and strategies that are observed in a lab task are in fact also used in coping with the complexity and ambiguity of the real-world task that the lab task is intended to reproduce and simplify.

Here, research on driving—and in particular driver gaze strategies—may be turned to, as a paradigmatic example to take as a model by researchers wishing to build toward an ecological approach (whether they are approaching it from the real world, building toward lab designs, or from lab paradigms, building toward ecological representativeness).

Eye movement research in driving has been able to reveal robust patterns that occur spontaneously in natural ecological conditions, without explicit instruction, or the need to develop novel, artificial stimuli to elicit them. The basic observations can be and have been reliably replicated. The behaviors can be reproduced and studied in driving simulators, and in the laboratory, where more experimental control is possible.

What can the gaze strategies tell us about the underlying perception, cognition, and control that is of general interest? Which of the gaze strategies reviewed here generalize to other environments can only be determined empirically. But the presence of essentially identical tracking fixations in walking (Matthis et al., 2018), and the fact that the visual pivot (and gaze anchoring) are strategies were first identified in teams sports (Klostermann et al., 2020; Vater et al., 2020), and look-ahead fixations in tea and sandwich making, hand-washing and block copying (Ballard et al., 1995; Land et al., 1999; Pelz and Canosa, 2001; Hayhoe et al., 2003; Mennie et al., 2007, see also Sullivan et al., 2021) suggest that when we *restrict* our viewpoint to driving and leave out of focus the complexity of “driver behaviors,” a *more general* picture emerges.

The lessons learned from eye movements should apply to other domains with similar task demands, and the ecological approach to driver behavior presented here should also work wherever a core subtask can be identified and extracted from the complexity of the real-world behavior it is embedded in (the more so to the extent the domain of interest shares the other useful features listed in Table 2).

This should not come as a surprise because the basic task of driving is, for the brain, simply the problem of visually guided high-speed motion in a 3D scene. To solve this task our brain most likely recycles pre-existing (evolved and learned) visual strategies and cognitive processes. In learning to drive, only the specific dynamic constraints and controls of a vehicle and recurring prototypical layout of the environments are “new”—but the brain does not need to (re)learn to “read” the environment, to find where free space or affordances for locomotion are. This is why the visual strategies and the underlying perceptual-cognitive mechanisms are quite general, and they involve no special mention of cars, roads, or the modern traffic system: while some visual strategies in driving might of course be specific tricks and heuristics for the modern road environment, others (especially those related to basic steering, speed and safety margin control) should be able reveal more general facts about visuomotor coordination, learning and skill development behind “the human advantage.”

Even though the remaining task is still complex, it occurs in an environment that constrains behavior sufficiently to produce experimental data that is feasible to do rigorous computational modeling on. There is still much need for developing rigorous models of the cognitive basis of the strategies—algorithmically implementable, quantitative process models that can yield a more mechanistic understanding of *what* information is gleaned *where* and *when*, in service of *which* computations. This is the way to a more principled interpretations of the function of the different types of fixations and saccades. In developing such models, it is critical that the models remain on a solid ecological footing, always with the goal of accounting for “genuine slabs of human behavior” (Newell, 1973 p.303), rather than just performance in a task that may not be ecologically representative.

### Conclusion

Driving is in many ways an attractive model behavior for developing ecologically representative task designs and studying real-world skills. Gaze behavior in driving also displays the seven “laws” that characterize general findings on naturalistic eye tracking research in the past 30 years (Lappi, 2016; Lappi et al., 2017; Table 2). But while this holds some promise for generality, it must be acknowledged that driver gaze behavior will undoubtedly a. include strategies that are specific to the driving task and b. not include strategies that may be widely spread among other natural tasks. Empirical research will be needed differentiate between general principles and task-specific cues and techniques, and only field research can reveal what strategies are actually used in which tasks in the real world.

And the seven “laws” themselves are of course themselves definitive—surely more “laws” remain to be discovered, in existing or future empirical data. Because human gaze control is flexible, many visual strategies could be possible in any task of ecologically representative task complexity and only experiment can show which ones are used. But taking an “ecological” approach does not imply rejecting lab work. It only means the laboratory must be like life. Field and lab studies are not alternatives, but complementary. While field research on naturalistic tasks is *necessary* it is not *sufficient*. And while rigorously controlled experimental task designs are *necessary*, they are not *sufficient* either.

Yet, the connection to real-world behavior should never be merely through the assumption that mechanisms studied in the lab are in principle “general.” This assumption is never warranted simply by the fact that the experimental task is simple and without domain-specific content—it is warranted only if there is an empirically sound case that it actually captures some relevant real-world processes in simplified form. The design of tasks and stimuli based on empirical knowledge of real behaviors as opposed to convenience of measurement, or for being computation-friendly. Only in this way can we hope to understand perception, cognition, and action that have adapted to the measurement-inconvenient and computation-unfriendly ecology of the real world.

## Author Contributions

The author confirms being the sole contributor of this work and has approved it for publication.

## Funding

During the preparation of this manuscript, the author was supported by the Academy of Finland (project grant 325694, UPP-PERFORMANCE, and fellowship 334192, Sense of Space). APC is covered from the research costs of the fellowship, (grant 336368).

## Conflict of Interest

The author declares that the research was conducted in the absence of any commercial or financial relationships that could be construed as a potential conflict of interest.

## Publisher’s Note

All claims expressed in this article are solely those of the authors and do not necessarily represent those of their affiliated organizations, or those of the publisher, the editors and the reviewers. Any product that may be evaluated in this article, or claim that may be made by its manufacturer, is not guaranteed or endorsed by the publisher.

## Footnotes

^1^ https://www.youtube.com/watch?v=wPrVM4sPY3k#t=3m51s
